# Patterns and seasonality of malaria transmission in the forest-savannah transitional zones of Ghana

**DOI:** 10.1186/1475-2875-9-314

**Published:** 2010-11-07

**Authors:** Dominic B Dery, Charles Brown, Kwaku Poku Asante, Mohammed Adams, David Dosoo, Seeba Amenga-Etego, Mike Wilson, Daniel Chandramohan, Brian Greenwood, Seth Owusu-Agyei

**Affiliations:** 1 Kintampo Health Research Centre, Ghana Health Service, Ministry of Health, P. O. Box 200, Kintampo, Ghana; 2 Noguchi Memorial Institute for Medical Research, University of Ghana, Legon, Ghana; 3 Infectious Tropical Diseases Dept. London School of Hygiene & Tropical Medicine, UK

## Abstract

**Background:**

Knowledge of the local pattern of malaria transmission and the effect of season on transmission is essential for the planning and evaluation of malaria interventions. Therefore, entomological surveys were carried out in the forest-savannah transitional belt of Ghana (Kintampo) from November 2003 to November 2005 in preparation for drug and vaccine trials.

**Results:**

A total of 23,406 mosquitoes were caught from 919 traps over the two-year period (November 2003 to November 2005): 54.3% were *Culicines*, 36.2% *Anopheles funestus*, and 9.4% *Anopheles gambiae*. Infection rates with *Plasmodium falciparum *were 4.7% and 1.5% for *Anopheles gambiae *and *Anopheles funestus*, respectively. Entomological inoculation rates (EIRs) were 269 infective bites per person per year in the first year (November 2003-October 2004) and 231 the following year (November 2004-November 2005). Polymerase Chain Reaction (PCR) analysis detected only *Anopheles gambiae *s.s. Nineteen mosquitoes were tested by PCR in the wet season; 16 were S-molecular form, 2 M-molecular form and 1 hybrid (S/M). In the dry season, sixteen mosquitoes were tested; 11 S-molecular form, 2 M-molecular form and 3 S/M hybrids. The frequency of knock down resistance (*kdr*) genotypes F(R) was 0.60.

**Conclusion:**

The dynamics and seasonal abundance of malaria vectors in the Kintampo area was influenced by micro-ecology, rainfall and temperature patterns. Transmission patterns did not differ significantly between the two years (2004 and 2005) and both *Anopheles gambiae *and *Anopheles funestus *were identified as effective vectors. EIR estimates in 2004/2005 were between 231 and 269 infective bites per person per year. The information provided by the study will help in planning intensified malaria control activities as well as evaluating the impact of malaria interventions in the middle belt of Ghana.

## Background

Malaria remains a major public health threat in sub-Saharan Africa as the most efficient vector, *Anopheles gambiae *s.l, continues to adapt to humans [1] and is a complex of sibling species taxa, thus resulting in a high vectorial capacity. The complex consists of seven species that vary in their ability to transmit malaria [2]. Currently known sibling species within the complex include *An. gambiae s.s. Anopheles arabiensis, Anopheles melas, Anopheles merus, Anopheles quadrianulantus *(A and B) and *Anopheles bwambae*. Their distribution is associated with particular climatic zones and degrees of aridity [3,4]. In some areas of sub-Saharan Africa, where mosquitoes of the *Anopheles gambiae *complex are the most important vectors of malaria, individuals may receive up to 800 infective bites per person per year (*ib/p/y*) [5]. However, in other areas of Africa, *An. gambiae *is found together with *Anopheles funestus *and both vectors compete in terms of their importance as malaria vectors [6]

The *An. funestus *group consists of seven to ten morphologically difficult to distinguish sibling species. Among the group, *An. funestus *s.s is the most anthropophilic and efficient vector. More recently, chromosomal analysis in sympatric populations of this vector has led to provisional names of chromosomal forms such as Folonzo and Kiribina [7]. This vector has been extensively described in Navrongo in northern Ghana and in neighbouring Burkina Faso, where it is an efficient malaria vector [7,8].

The entomological inoculation rate (EIR) estimates the level of exposure of an individual to malaria-infected mosquitoes and it is the most favoured measure of assessing malaria endemicity and transmission intensity [5,9]. There is a strong correlation between EIR and the prevalence of malaria in a population and, as such, it has become the most accurate measure for estimating transmission. Estimated EIR in the northern part of Ghana (Navrongo), where there is an irrigation programme, is 643 infective bites per person per year (*ib/p/y*) [8]. In the southern forest (Dodowa) and coastal areas (Prampram) of Ghana, estimated EIRs were 21.9 and 3.65 respectively [4]. There is, however, little information about the intensity of malaria transmission in the middle belt of Ghana required for the design of effective vector control strategies.

*Anopheles gambiae s.s*. has been shown by the extent of chromosomal inversion polymorphism and, more recently, by divergence at the molecular level to consist of two molecular forms M and S [3]. In addition, five chromosomal forms named under a non-linean nomenclature as Forest, Savanna, Mopti, Bamako and Bissau have been identified [10]. This vector has been extensively implicated in malaria transmission in West Africa and Ghana in particular [11]. Climate affects the distribution of both chromosomal and molecular forms [3].

Insecticide resistance of the type *kdr *(knock down resistance), which influences response to pyrethroids and DDT, has been detected in many West African populations of *An. gambiae *s.s [12]. Mutations in the *kdr *target site, voltage gated sodium channel, have been observed in West Africa *An. gambiae *species [13]. In most investigated West Africa countries, the *kdr *allele was detected in S populations and absent in all sympatric M populations, thus supporting the hypothesis of reduction of gene flow between them [12]. Data on *An.gambiae *s.s indicate introgression in the S and M molecular forms in Benin and countries to the east [14]. The *kdr *allele is observed in the M form in Benin [2], but mainly in the S form in most West African countries such as Ivory Coast and Ghana, Nigeria, Mali and Burkina Faso [1,10,12,15,16]. Though at a lower frequency, *kdr *resistance has also been reported in the M form in Ghana and Burkina Faso [11,15].

This entomological survey was designed to answer basic entomological questions concerning the transmission of malaria within the forest-savannah transitional zone in Ghana to serve as baseline work for monitoring transmission dynamics and the impact of malaria interventions in this area.

## Methods

### Study area, selection of communities and meteorological data

Kintampo North and South districts lie within the forest-savannah transitional ecological belt in the Brong Ahafo region of Ghana. They cover an area of approximately 7,162 km^2 ^with a resident population of approximately 140,000 living in approximately 22,000 houses in 156 villages. Communities in Kintampo are predominantly agricultural, engaging in farming of maize and yam and raising some livestock. The area has a mean monthly temperature range of 18°C to 38°C and a rainfall averaging 1,250 mm per annum, which falls mainly between April and October. The Kintampo Health Research Centre implements a health and demographic surveillance system (KHDSS), which tracks the resident population dynamics every six months [17].

Sixteen communities or clusters were randomly selected for inclusion in this study following stratification reflecting the mixed micro-ecology (based mainly on the vegetation and water bodies) of the two districts. Communities were geo-located using a simple hand-held GPS receiver (GARMIN series) and integrated into a Geographic Information System (GIS) for analysis (ArcGIS 9.2 version). Every house within clusters or communities was given an equal chance of participation. Selection of houses to receive light traps was done without replacement until all houses within a particular community had been considered.

One of the meteorological stations linked to the Ghana National Meteorological Department is located in Kintampo town and serves the two districts. Daily collection of data (including rainfall data) was performed for the entire period of the survey.

### Mosquito collection and identification

Mosquito collection was performed over a two-year period (November 2003 to November 2005), the first year alongside a parasitological survey and the second year alone. The Centre for Disease Control (CDC) light traps were used to collect mosquitoes in rooms of randomly selected households. In the first year, mosquito trapping was undertaken the night before a parasitological survey in a particular cluster. Additional traps were then set weekly in rooms of study subjects who met selection criteria in both cohort studies [18] ensuring that at least one trapping took place in each month in each of the 16 selected communities and throughout the whole of the first year of the study. Verbal consent was sought from household heads and occupants of each room in which a trap was set and untreated bed nets provided to be used during the night that the trap was set in the room. Traps were hung approximately 1.5 m above the floor at the foot of the bed/mat of the index person. In the second year of mosquito collection (December 2004 to November 2005), the number of CDC light traps was reduced from four per community per night to two per community per night of trapping due to funding limitation. All other processes were maintained as during the preceding year.

*Anopheline *vectors were morphologically identified into species using identification keys [19], stored in 1.5 ml micro-centrifuge tubes enclosed in zip lock plastic bags with silica gel. A maximum of 10 mosquitoes of the same species from the same compound was put in a tube. Non-*Anopheles *species were discarded after recording numbers caught.

### Circumsporozoite enzyme-linked immunosorbent assay (CS-ELISA)

Heads and thoraces of the two major vectors of malaria, *An. gambiae *and *An. funestus*, were checked for the presence of circumsporozoite (CS) antigen of *P. falciparum *using the sandwich enzyme-linked immunosorbent assay (ELISA), as described [20]. Presence of CSP in the mosquitoes was read at 405 nm wavelength using a micro plate ELISA reader. A cut-off of 0.2 nm absorbance after subtraction of the average value from seven negative test mosquitoes was considered positive. Heads and thoraces of male *Anopheles *vectors were used as the negative test controls. All positive mosquitoes were retested to confirm positivity. Results were analysed with the aid of Pool Screen^® ^computer software. EIR was calculated by multiplying the proportion of positive tested vector species by their Man Biting Rate (MBR), which is estimated as the geometric mean of the number of vectors caught in a light trap.

### Species identification, molecular form and *kdr *genotyping

A total of sixty-four sub-samples were randomly selected to explore the most predominant specie within *An. gambiae *complex. The samples were selected from the month of February (to represent dry season) and August (to represent wet season) in 2005 catches of *Anopheles *and tested by Polymerase Chain Reaction (PCR) as per protocol [21]. Enzyme digestion to differentiate the molecular forms of *An. gambiae s.s *was performed as per protocol [22] on samples that were first PCR successful for sibling species analysis. The presence of *kdr *alleles conferring knock-down resistance in West Africa was assessed as described [23] on samples that were PCR successful for molecular form differentiation analysis. Results were analyzed proportionally and by Hardy-Weinberg test statistic.

## Results

### Mosquito abundance, parasite prevalence and rainfall in Kintampo district

**Figure 1:**
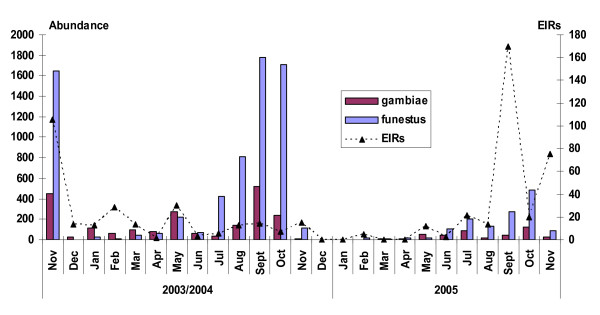
**Monthly vector abundance and EIRs in Kintampo (2003 - 2005)**.

**Figure 2:**
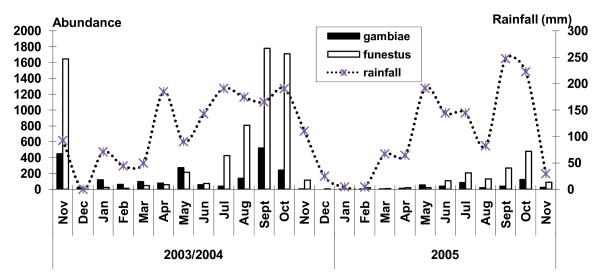
**Monthly vector abundance and rainfall in Kintampo (2003 - 2005)**.

In year 1 (November 2003 - November 2004), 664 CDC light traps caught 19,771 mosquitoes; 51.5% of these were *Culicines*, 1.0% *Aedes*, 35.0% *An. funestus*, 10.6% *An. gambiae *and 1.9% *Anopheles rufipes*. In year 2 (December 2004-November 2005), 355 CDC traps set captured 3,571 mosquitoes; 44.1% of these were *Culicines*, 4.8% *Aedes*, 37.9% *An.funestus*, 11.6% *An.gambiae *and 1.6% *An. rufipes *(Table 1). Monthly abundance of *Anopheles *vectors caught varied in the two collection periods. *Anopheles funestus *was caught most frequently in November 2003, September 2004 and October 2005. *Anopheles gambiae *was also caught frequently in these months but in lower numbers than *An. funestus *(Figure 1). Abundance of both vectors correlated with monthly rainfall patterns (Figure 2). The annual rainfall in Kintampo for three years consecutively (2003-2005) was steady and ranged between 1,200 mm and 1,400 mm. The highest rainfalls were recorded in the months of April, July and September in the first year and in May, September and October in the second year (Figure 2).

**Table 1:** Mosquito densities in Kintampo from CDC light trap catches

PERIOD (Nov 2003 - Nov 2004)					
	***An. gambiae***	***An. funestus***	***An. rufipes***	***Culex***	***Aedes***

Mean catch per night	3.16	10.42	0.56	15.33	0.30
Maximum per catch	129	1,040	42	1,311	36
**Total (19,771)**^**a**^	**2099**	**6922**	**370**	**10178**	**202**
**%**	**10.62**	**35.01**	**1.87**	**51.48**	**1.02**

**PERIOD (Dec 2004 - Nov 2005)**					

	*An. gambiae*	*An. funestus*	*An. rufipes*	*Culex*	*Aedes*

Mean catch per night	1.16	3.81	0.16	4.44	0.49
Maximum per catch	64	360	14	247	102
**Total (3,571)**^**b**^	**413**	**1352**	**57**	**1576**	**173**
**%**	**11.57**	**37.86**	**1.60**	**44.13**	**4.84**

Superscripts ^a, b ^are totals of all species of mosquito collections in (2003-2004) and (2004-2005) respectively

**Figure 3:**
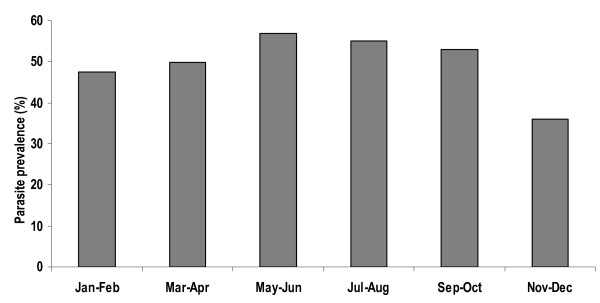
**Bimonthly *P. falciparum *parasite *prevalence in *all-age groups in Kintampo**.

The all-age group prevalence of *Plasmodium falciparum *ranged between 36% and 57%. The prevalence was highest during the rainy seasons (May-October) but high throughout the year (Figure 3).

**Figure 4:**
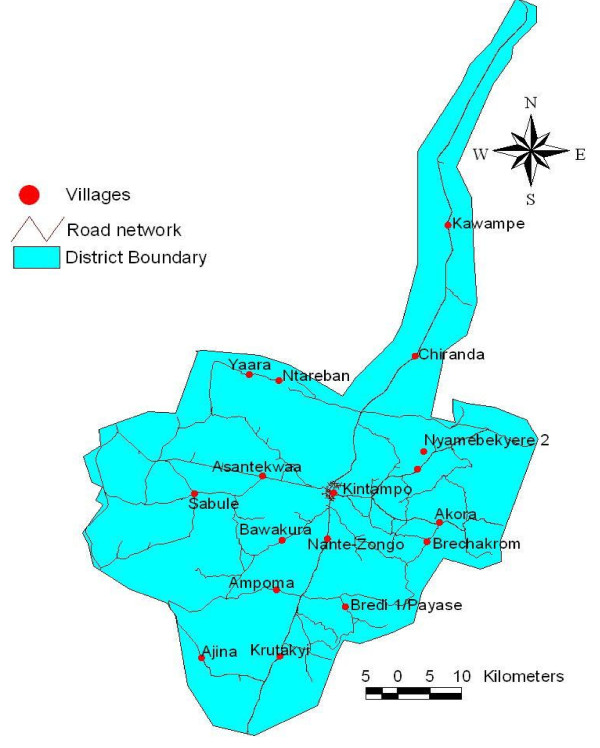
**Map of surveyed communities with distances in Kintampo (2003 - 2005)**.

The distances of communities the study was carried out ranged between eight kilometres and 50 kilometres from the meteorological station centrally located in the two districts. The farthest community to the extreme south (Ajina), is 46 km and that to the extreme north (Kawampe), is 50 km (Figure 4).

### Infectivity and seasonal transmission of the main malaria vectors

The two main malaria vectors were *An. gambiae *s.s and *An. funestus*. In year one, 8,418 *Anopheline *samples were assayed by CS-ELISA; 6,542 were *An.s funestus *and 1,876 were *An. gambiae. Plasmodium falciparum *CS antigen positivity was 1.5% in *An. funestus *and 4.7% for *An. gambiae*. The annual EIR, including both vector species, was 269 infective bites per person per year *(ib/p/y) *with variations in community level EIRs. A total of 1,794 *Anopheline *samples were tested by CS-ELISA in year two; 1,054 were *Anopheles funestus *and 740 were *An. gambiae. Plasmodium falciparum *CS antigen positivity was 3.7% in *An. funestus *compared to 1.2% for *An. gambiae*. The annual EIR was 231*ib/p/y *with *An. funestus *(141*ib/p/y*) contributing more infective bites than *An. gambiae *(90*ib/p/y*), the opposite pattern from that observed in the previous year. Sporozoite rates (SRs) were generally higher in year 2 than in year 1. Sporozoite rates were highest in communities in the north east and in the southern parts of the districts (Table 2).

**Table 2:** Sporozoite rates (*SR*) in surveyed communities in Kintampo.

	Year 1			Year 2		
**Community**	***Test***	***Pos***	***SR***	***Test***	***Pos***	***SR***

Kintampo town	60	0	0	28	3	0.107
Ajina	60	2	0.033	8	1	0.125
Asantekwa	472	8	0.017	26	3	0.115
Nyame.2, Brecha	70	1	0.014	27	0	0.000
Bredi	75	0	0	25	7	0.280
Bawakura	181	1	0.006	41	2	0.049
Ntereban	2,248	71	0.032	187	17	0.091
Chiranda	1,569	22	0.014	926	35	0.038
Kawampe	1,377	16	0.012	70	4	0.057
Krutakyi	28	1	0.036	23	1	0.043
Sabule	274	7	0.026	165	22	0.133
Nyame.No1	98	1	0.010	10	0	0.000
Ampoma	57	1	0.018	90	30	0.333
Nante-Zongo	32	0	0	78	7	0.090
Yara	1,455	50	0.034	90	4	0.044
Akora	130	4	0.031	0	0	0.000

**Total**	**8186**	**185**	**0.023**	**1794**	**136**	**0.076**

*Pos *- sporozoite positive by ELISA; *SR *- Sporozoite Rate; *Test *- *Anopheles *tested

### Species, molecular forms and knock-down resistance (*kdr*)

Fifty-five out of the 64 selected *An. gambiae s.l*. were successfully tested by polymerase chain reaction (PCR) to determine their sub-species. All were *An. gambiae *s.s; no *An. arabiensis *were detected in either survey period. Sixteen of 22 *An. gambiae s.s*. mosquitoes collected during the wet season (August 2005) were S molecular form, two M molecular forms and one a hybrid (S/M). Three samples failed PCR analysis. A similar pattern was seen during the dry season (February 2005) with 11 of 16 *An. gambiae s.s*. mosquitoes tested being S molecular form, two M molecular form and three (S/M) hybrids (Table 3). Ten *An. gambiae s.s *mosquitoes, five collected in the rainy season and five collected in the dry season were tested for *kdr *mutations (Table 3). Six were *kdr*^RR^, two *kdr*^R/S ^and two had the susceptible *kdr*^ss ^genetotypes.

**Table 3:** Species, molecular forms and *kdr *resistance in Kintampo

		**Species**		**Molecular forms**			***kdr *mutation**			
				
**Area**	**season**	**Ag**.	**Ar**.	**M**	**M/S**	**S**	***kdr***^**RR**^	***kdr***^**R/S**^	***kdr***^**SS**^	**F(R)**
		
	***Wet***	22	0	2	1	16	3	1	1	
				
**Kintampo**	***Dry***	33	0	2	3	11	3	1	1	**0.60**

Hardy-Weinberg expected frequencies not significantly different (P < 0.05; df = 1; χ^2 ^= 0.005)

Wet and Dry represent raining/wet season and dry season respectively

## Discussion

This study has demonstrated a high level of malaria transmission in the middle belt of Ghana with each individual in the study area receiving between 231 and 269 infective bites in each year and, during the peak transmission season, one bite every day by a mosquito infected with *P. falciparum*. There were differences in the densities of vector caught in the various communities. Communities to the north-west (Yara, Ntereban and Sabule) had high densities of *Anopheles *vectors caught monthly [18]. Variations in transmission between communities reflect the importance of micro-ecological factors in the area studied. Mosquito abundance was greatly influenced by rainfall. Transmission in the Kintampo area (231-269 *ib/p/y*) is lower than that recorded in the Navrongo area in northern Ghana [8], but significantly higher than that recorded in Dodowa and Prampram near the coast [4]. This is most probably due to the mixed forest-savannah micro-ecology in the middle belt of Ghana.

The two main malaria vectors in the Kintampo area are *An. gambiae *and *An. funestus *with their abundance depending on the season. *Anopheles gambiae *was the main vector in 2003/2004, whilst *An. funestus *became the main one in 2005. This is supported by studies [6,24] that indicate the obvious importance of these species as the major malaria vectors in Africa. Transmission was sustained throughout the year despite variations in inoculation rates in the dry and wet season. The dramatic increase in numbers of *Anopheline *vectors observed at the onset of the rains in both years was not translated into an increase in EIRs in these months because sporozoite rates were low, presumably reflecting low infectiousness of the human reservoir [25]. Also at the beginning of the rainy season, there is high abundance of relatively young vector populations which are less likely to be infective and are easily attracted to light traps. Largely, rainfall patterns correlated with high densities of vectors caught per month in the communities though in the first year this was not very obvious for all the months; a demonstration of the impact of environmental factors (weather) on vector abundance, distribution and malaria transmission [26].

The results of this study suggest that the most prevalent molecular form within *An gambiae *s.s in the Kintampo area is the S form although this conclusion is made with lots of caution as very limited numbers of samples were successfully analyzed by PCR. Nonetheless, this observation supports studies [10,12,27] that have shown that the M form is most prevalent in drier environments where breeding takes place all year round due to activities such as irrigated projects while the S form is exclusively found in more humid, forested areas. The mixed micro-ecology in the Kintampo area probably accounts for the occurrence of both the S and M forms and, therefore, supports [28] which observed the S form in the middle areas of Ghana and M form in northern savannah and coastal areas of the country. The presence of hybrids in both the dry and wet seasons suggests the overlap of niches of these sub-species and the possibility of interbreeding and perhaps gradual gene flow within the complex. This supports the hypothesis that hybrids are viable in natural populations with no evidence of reduced fitness [15].

The frequency of *kdr *resistant genotypes F(R) in the Kintampo area is lower than that found in neighbouring countries such Benin and Togo [29,30]. Field resistance susceptibility bioassays are needed to support the picture indicated by these molecular studies. These limited observations cause concern about the impact of *kdr *resistance, and of other forms of resistance in malaria vectors which have not been studied in this study area but reported in northern Ghana (Navrongo) [31], on the long-term efficacy of pyrethroid based nets and other materials in this part of Ghana.

## Conclusions

Malaria transmission in the Kintampo area of the middle belt of Ghana, studied in 2004 and 2005, is high and occurs all-year round. The intensification of malaria control activities; introduction of artemisinin-based combination treatment (ACT), distribution of ITNs for under five year olds, in the past five years may have altered the situation requiring the repeat of similar studies in order to determine the impact of existing control measures have had on malaria transmission in the middle belt of Ghana and how this contributes to the malaria elimination/eradication discussions currently ongoing.

## Competing interests

The authors declare that they have no competing interests.

## Authors' contributions

SOA conceptualised the idea, secured funding and was the principal investigator. MA and DD carried out sample collections; SAE performed data management; DD, CB and DO carried out laboratory analyses; DD and CB drafted the initial paper; KPA and SOA supervised data collection, analyses interpretation and write up and MW, DC, BG advised on the design and implementation of the study and also reviewed and finalised this paper for publication. All authors read and approved the final manuscript.
